# Predicting Breast Tumor Malignancy Using Deep ConvNeXt Radiomics and Quality-Based Score Pooling in Ultrasound Sequences

**DOI:** 10.3390/diagnostics12051053

**Published:** 2022-04-22

**Authors:** Mohamed A. Hassanien, Vivek Kumar Singh, Domenec Puig, Mohamed Abdel-Nasser

**Affiliations:** 1 Department of Computer Engineering and Mathematics, Univerity Rovira i Virgili, 43007 Tarragona, Spain; mohamed.abdelhameedhassanien@estudiants.urv.cat (M.A.H.); domenec.puig@urv.cat (D.P.); m.nasser@ieee.org (M.A.-N.); 2 Precision Medicine Centre of Excellence, School of Medicine, Dentistry and Biomedical Sciences, Queen’s University Belfast, Belfast BT7 1NN, UK; 3 Electrical Engineering Department, Aswan University, Aswan 81528, Egypt

**Keywords:** breast cancer, CAD system, ultrasound sequence, deep learning, transformers

## Abstract

Breast cancer needs to be detected early to reduce mortality rate. Ultrasound imaging (US) could significantly enhance diagnosing cases with dense breasts. Most of the existing computer-aided diagnosis (CAD) systems employ a single ultrasound image for the breast tumor to extract features to classify it as benign or malignant. However, the accuracy of such CAD system is limited due to the large tumor size and shape variation, irregular and ambiguous tumor boundaries, and low signal-to-noise ratio in ultrasound images due to their noisy nature and the significant similarity between normal and abnormal tissues. To handle these issues, we propose a deep-learning-based radiomics method based on breast US sequences in this paper. The proposed approach involves three main components: radiomic features extraction based on a deep learning network, so-called ConvNeXt, a malignancy score pooling mechanism, and visual interpretations. Specifically, we employ the ConvNeXt network, a deep convolutional neural network (CNN) trained using the vision transformer style. We also propose an efficient pooling mechanism to fuse the malignancy scores of each breast US sequence frame based on image-quality statistics. The ablation study and experimental results demonstrate that our method achieves competitive results compared to other CNN-based methods.

## 1. Introduction

According to WHO (World Health Organization) reports, breast cancer is one of the most common cancers in women worldwide (https://www.who.int/news-room/fact-sheets/detail/breast-cancer accessed on 1 April 2022). The malignant growth of BC begins within the duct or lobule, where it usually does not cause symptoms and has a low risk of extending to other body parts (i.e., metastasis). In situ breast tumors can grow and intrude into surrounding breast tissue, then spread to nearby lymph nodes or other organs (i.e., distant metastasis). It is worth noting that widespread metastasis is the leading cause of death in breast cancer patients [1]. Hence, breast cancer must be detected early to reduce mortality. Many countries across the world have developed some prevention programs that perform routine screening for women.

The presently used clinical breast imaging modalities are mammography, magnetic resonance imaging (MRI), and ultrasound imaging (US). Currently, MRI and US are only auxiliaries to mammography. Mammography imaging sensitivity is approximately 75%, which can be dropped to 50% in young women whose breast tissues frequently have a higher breast density [2]. Hence, the use of mammography and US imaging could significantly enhance the sensitivity of the test for the diagnosis of such cases [3]. Unlike other imaging modalities, such as MRI, breast ultrasound (BUS) technology is much cheaper, fast, and easily accessible to people in the community. BUS imaging offers scanning feasibility to women who are at high risk of breast cancer disease. BUS imaging supports women during their pregnancy without them being exposed to radiation. However, during BUS scanning, some artifacts are produced due to the motion of the sonographer, patient breathing, and poor probe contact that cause a poor image formation on-screen [4].

Indeed, an experienced sonographer is required to extract and interpret tumor information from BUS images. Given the number of ultrasound images a sonographer must analyze, this is time-consuming and costly. In this situation, a computer-aided diagnosis (CAD) system can relieve professional sonographers’ burden by providing helpful diagnostic clues such as the likely location of tumors, their plausible borders, and a prediction of tumor type [5]. Because the manual diagnosis of breast cancer takes a long time and limited detection technologies are available, an automatic diagnosis system is needed for early cancer detection.

**Figure 1:**
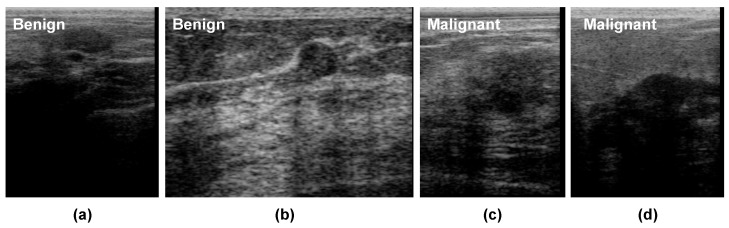
Examples of BUS images for (**a**,**b**) benign and (**c**,**d**) malignant cases. They show the presence of artifacts such as speckle noise and the hypoechoic region with shadows caused by the reflection of a high quantity of energy by an enormous impedance discontinuity and a variety of breast tumor shapes and sizes.

Figure 1 presents some BUS images of benign and malignant tumors. As shown, it is challenging to analyze breast tumors in BUS images due to their low contrast, poor signal-to-noise ratio (SNR), the great shape variety of breast tumors, and the hazy nature of BUS images. Tumor segmentation and classification are two crucial tasks in CAD systems. Benign and malignant tumors usually display different visual characteristics in BUS images. The margins of most benign tumors are smooth, round, or oval, but the borders of most malignant tumors are irregular and spiculated [6]. Nevertheless, designing CAD systems for BUS is still challenging due to the large variation in tumor size and shape, ambiguous tumor boundaries, and low SNR.

Deep learning has improved the automated analysis of BUS images in the last decade, thanks to its ability to extract powerful representations from them. Hence, several deep-learning-based CAD systems have been proposed to detect breast cancer or discriminate between benign and malignant tumors [7]. For instance, Masud et al. [8] used ultrasound images to develop and assess three pretrained convolutional neural network (CNN)-based models for recognizing breast cancer. The authors tweaked AlexNet [9], DenseNet121 [10], MobileNetV2 [11], ResNet-18 [12], ResNet-50 [12], VGG16 [13], and Xception’s [14] pretrained models to extract powerful representative features from BUS and to add a classifier to the top layer. Most existing studies employ a single ultrasound image (SUI) for each breast tumor to extract features to discriminate between benign and malignant tumors. However, artifacts in BUS images such as speckle noise and shadows (as shown in Figure 1) may degrade the performance of feature extraction methods. Unlike most existing SUI-based studies, we propose to use deep-learning-based radiomic features extracted from BUS sequences in this paper. Specifically, we employ the ConvNeXt [15] network, which is a CNN trained using the vision transformer style. The proposed approach contains three main components: radiomic features extraction based on ConvNeXt, malignancy score pooling mechanism, and visual interpretations.

The key contributions of this paper can be listed as follows:

- Propose an efficient deep-learning-based radiomics method to predict a malignancy score for breast tumors from BUS sequences.
- Propose an efficient malignancy score pooling mechanism for BUS sequences, in which the quality of each frame in the input BUS sequence is assessed to compute its weight when calculating the overall malignancy score.
- Provide comparisons between CNN-based radiomics and transformer-based radiomics approaches.
- Present visual interpretations for the decisions of the proposed ConvNeXt-based radiomics approach.

The rest of this paper presents and discusses the state-of-the-art methods in Section 2 and the proposed approach for predicting breast cancer malignancy in BUS images in Section 3. The evaluation of the proposed method and discussion of the results are provided in Section 4. The conclusion of the study and lines for future studies are presented in Section 5.

## 2. Related Work

Most CAD systems in the literature employ a single ultrasound image (SUI) for each breast tumor to classify it as benign or malignant. Table 1 presents and summarizes different related studies. In [16], an automatic thyroid and breast lesions classification method from ultrasound images using deep CNNs was proposed. A generic deep learning architecture with transfer learning and the same architectural parameter settings to train models for thyroid (TNet) and breast cancers (TNet and BNet) was presented. The authors achieved accuracy rates lower than 90% with both tasks in ultrasound images collected from clinical practices. Pourasad et al. [17] compared the performance of six traditional and deep-learning-based systems for detecting and segmenting tumors in BUS images. In the case of conventional systems, they used the fractal method to select features, and the K-nearest neighbor (KNN), support vector machine (SVM), decision tree (DT), and Naïve Bayes (NB) classification techniques were used to classify images into normal, benign, and malignant. In turns, a deep-learning-based system was used with a CNN architecture to classify BUS images. This method obtained a limited sensitivity of 88.5% and depended on many preprocessing techniques that should be tuned to reach a good accuracy with each new dataset.

Jabeen et al. [18] proposed a deep-learning-based CAD system for breast cancer classification in BUS images. The authors modified a pretrained DarkNet53 model and trained it on augmented BUS images using transfer learning. They experimented with the CAD system using a dataset of 780 samples (133 normal, 210 malignant, and 487 benign). Cao et al. [19] proposed a CAD system that included a tumor detection stage followed by a tumor classification stage to classify breast tumors as benign and malignant from BUS images. In the tumor detection stage, they evaluated five deep-learning-based object detection methods, namely, fast region-based convolutional neural networks (R-CNN), faster R-CNN, you only look once (YOLO), YOLO version 3 (YOLOv3), and single shot multibox detector (SSD). In the tumor classification stage, they evaluated six CNN architectures, namely AlexNet, ZFNet, VGG, ResNet, GoogLeNet, and DenseNet, with different model training parameters values in classifying breast tumors as benign or malignant. The authors collected a BUS images dataset containing 579 benign and 464 malignant cases. With this dataset, DenseNet achieved the best classification results with an accuracy of 87.5%. It is worth noting that the main limitation of this method is that some tumors may be missed because of the detection step, which had a low F1-score of 79.38%.

Luo et al. [20] proposed a segmentation-to-classification method by adding the segmen tation-based attention information to the breast tumor classification network. Their method comprised four stages. First, the segmentation network was trained to segment breast tumors from BUS images. Second, the authors used two parallel networks to extract features from the original BUS images and segmented ones. Third, they used a channel-attention-based feature aggregation network to fuse the features extracted from two feature networks. Finally, the fused features were fed into a classification network to discriminate between malignant and benign tumors. With a private breast ultrasound dataset, the authors obtained an AUC of 95.49%. Zhou et al. [21] proposed a multitask deep-learning-based method to jointly train breast tumor segmentation and classification network for a 3D automated breast ultrasound (ABUS). The proposed network included an encoder–decoder network for segmentation and a lightweight multiscale network for classification. The authors employed VNet as the backbone network for tumor classification and segmentation. With a private dataset of 170 volumes from 107 patients, they achieved an accuracy of 74.10% when classifying benign and malignant cases. The main limitations of this study are that (1) the failures in the segmentation part affect the final classification results and (2) the performance of the deep learning network may be degraded because of data imbalance.

Furthermore, Mishra et al. [22] proposed a machine-learning-based radiomics approach to classify breast ultrasound images into benign and malignant. The authors utilized the ground truth of the database to segment the tumor region and then extracted a set of handcrafted features (i.e., histogram of oriented gradients, gray lever co-occurrence matrix features, shape features, and Hu moments). A recursive feature-elimination-based feature selection step was used to select the best features and a synthetic minority oversampling technique (SMOTE) to deal with the data imbalance problem. Finally, different classifiers were evaluated in the classification step. Hassan et al. [23] investigated a semisupervised generative adversarial network (GAN)-based approach to augment imaging datasets for breast tumor classification on ultrasound images. The authors used a semisupervised GAN network called TripleGAN to synthesize the textural patterns of breast tumors. The proposed approach performed preprocessing steps, in which feature-wise processing (FWP) was applied to reduce the deep learning model processing time on raw ultrasound images. The images were cropped to 128×128 pixels as the regions of interest (ROI). The real and synthesized image were fed into an Inception-V3 model to classify BUS images into benign and malignant. On a private dataset that included ultrasound images of 767 benign and 680 malignant tumors the authors obtained a 90.4% accuracy, an 87.94% sensitivity, and an 85.86% specificity.

As discussed above, most studies did not consider the quality of BUS images when building the classification models. Furthermore, they employed a single BUS image to develop their methods. However, the noisy nature of BUS images and the significant similarity between normal and abnormal tissues make them difficult to recognize, causing incorrect diagnosis. In addition, dense breast fat and glandular tissue produce attenuation that affects ultrasonic waves and consequently degrades image quality. These issues represent a challenge to build a robust BUS image classification model. To handle these issues, this paper proposes an effective deep-learning-based radiomics method for breast cancer malignancy prediction from BUS sequences. To extract robust breast-tumor-relevant representations, we employ a deep learning architecture called ConvNeXt network. Unlike most existing work that employed a single BUS image for each tumor to build the classification model (i.e., SUI CAD system), we utilize BUS sequences. We also propose a malignancy score pooling mechanism that considers the BUS image quality when computing the final malignancy score of the whole sequence.

## 3. Methods and Materials

**Figure 2:**
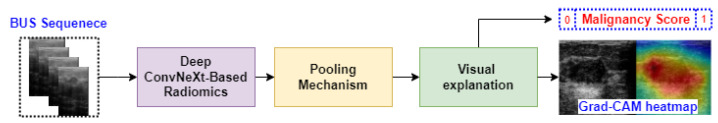
Overview of deep ConvNeXt-based radiomics for breast tumor malignancy prediction from BUS sequences.

Figure 2 presents an overview of the proposed approach for predicting breast cancer malignancy from BUS images. As shown, the proposed method comprises three main components: (1) an emerging deep learning network called ConvNeXt [15] to extract robust radiomic features, (2) a pooling mechanism to generate the malignancy score of each input BUS sequence, and (3) a visual explanation algorithm to help interpret deep learning decisions. Three components of the proposed method are illustrated below in detail.

### 3.1. Deep ConvNeXt-Based Radiomics

**Figure 3:**
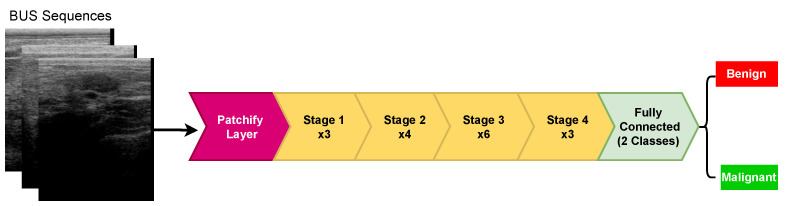
Schematic diagram of ConvNeXt. It should be noted that ×3, ×4, ×6, and ×3 mean that there are 3, 4, 6, and 3 blocks in stage 1, 2, 3, and 4, respectively.

We employed ConvNeXt [15] to extract robust radiomic features to classify breast cancer tumors as benign or malignant. In ConvNeXt, the architecture of the standard CNNs is modernized to the construction of a hierarchical vision transformer. As discussed in [15], the starting point of ConvNeXt is a ResNet-50 [12] model, which has four stages, each containing several blocks. In ConvNeXt, the ResNet-50 model has been trained with similar training techniques used to train vision transformers. As shown in Figure 3, ConvNeXt is a multistage design with varying feature map resolutions for each stage, in which the stage-compute-ratio—SCR (number of blocks per stage)—and stem cell structure are the two design concerns. ConvNeXt has four stages, where the SCR is set to (3, 4, 6, 3). ConvNeXt employs a *patchify* layer implemented using a 4×4, stride 4 convolutional layer. The patchify layer is a distinct difference between ConvNeXt and ResNet (and CNNs in general), which uses a stem cell comprising a 7×7 convolution layer with stride 2, followed by a max-pool.

**Figure 4:**
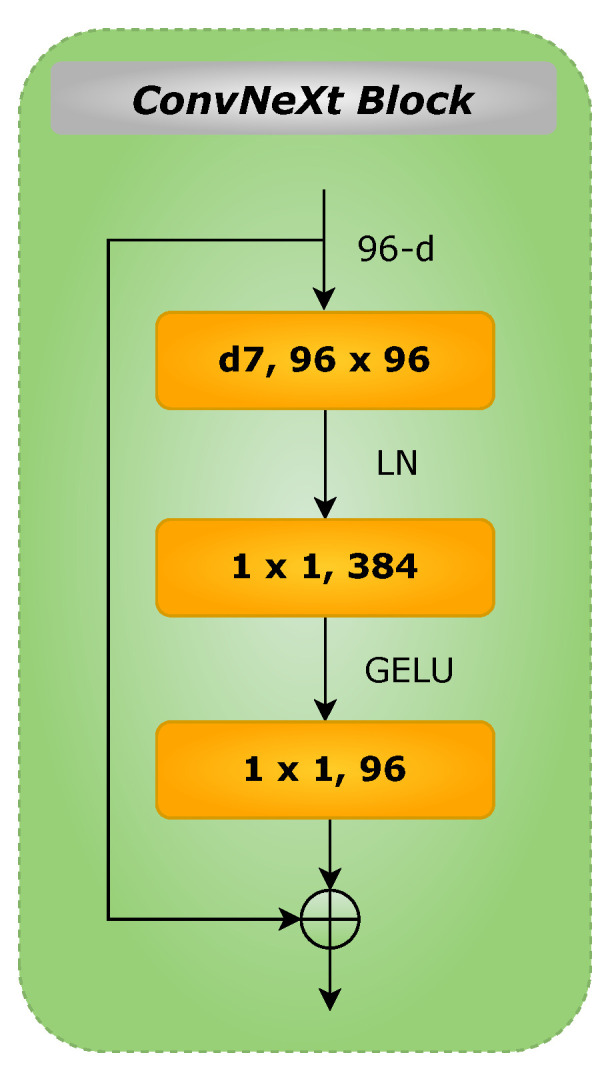
Schematic diagram of ConvNeXt block.

Figure 4 depicts the schematic diagram of ConvNeXt block. As shown, the block contains a 7×7 depthwise convolution, two 1×1 layers, and a nonlinear GELU activation (gaussian error linear unit, a smoother variant of ReLU). Layer normalization (LayerNorm) is used before the Conv 1×1 layer. For an input *z*, GELU can be expressed as follows [24]:

(1) $$\mathrm{GELU}\left(z\right)=zP\left(Z\leq z\right)=z\Phi \left(z\right)=z\cdot \frac{1}{2}\left[1+\mathrm{erf}\left(z/\sqrt{2}\right)\right]$$

It should be noted that the GELU expression mentioned in (1) can be approximated as follows [24]:

(2) $$0.5x\left(1+\tanh\left[\sqrt{2/\pi }\left(x+0.044715x^{3}\right)\right]\right)$$

In the ConvNeXt model, the LayerNorm method is used to avoid the disadvantages of the batch normalization technique widely adopted in existing deep CNN architectures (e.g., computational cost and discrepancy between training and inference). Considering that changes in one layer’s output will tend to produce strongly correlated changes in the total inputs to the next layer, by setting the mean (μ) and variance (σ) of the summed inputs inside each layer, LayerNorm eliminates the *covariate shift* problem. The LayerNorm statistics are calculated as follows for all hidden units in the same layer [25]:

(3) $$\mu^{l}=\frac{1}{H}\sum_{i=1}^{H}a_{i}^{l}\;\sigma^{l}=\sqrt{\frac{1}{H}\sum_{i=1}^{H}{\left(a_{i}^{l}-\mu^{l}\right)}^{2}}$$

where *H* stands for the number of hidden units in a layer in this formula. It is worth noting that LayerNorm has no restrictions on the size of a minibatch and can be utilized in the pure online mode with batch sizes as small as one.

Furthermore, the ConvNeXt architecture utilizes depthwise convolution, a type of grouped convolution in which the number of groups and channels is equal. Indeed, depthwise convolution is analogous to the per-channel weighted sum operation in the self-attention mechanism (mixing information in the spatial dimension). ConvNeXt adds a separate downsampling layer between stages. It uses 2×2 Conv layers for downsampling with a stride of 2. In this work, we used the cross-entropy (CE) loss function to train the model. CE can be expressed as follows [26]:

(4) $$L^{\mathrm{CE}}\left(gt,p_{i}\right)=-\sum_{i=1}^{n}gt.log\left(p_{i}\right)$$

where *n* corresponds to the number of classes, gt is the ground truth label, and $p_{i}$ refers to the softmax probability of the *i*th class.

In the training phase of the ConvNeXt model, we rescaled the original BUS input resolution to a size of 224×224. An ADAM optimizer with $\beta_{1}$ = 0.5, $\beta_{2}$ = 0.99, and a starting learning rate of 0.0001 were utilized to optimize the model nicely. We employed a batch size of two images and trained the model for 40 epochs. All the models were developed using Python on PyTorch with an NVIDIA GeForce GTX 1070Ti GPU with 8 GB RAM.

### 3.2. Malignancy Score Pooling Mechanism

**Figure 5:**
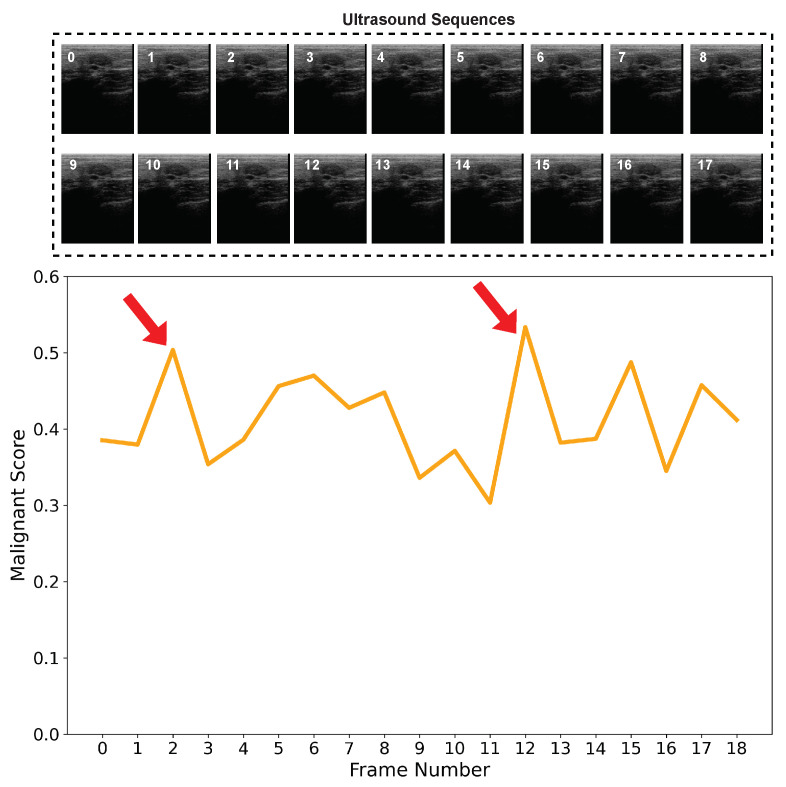
Malignancy score of each frame in a BUS sequence of a benign case. Arrows indicate malignancy scores higher than 0.5.

Most of the existing methods extract radiomic features from a single BUS image. BUS image artifacts such as speckle noise and shadow may degrade the performance of the extracted radiomic features and yield wrong classification results. In Figure 5, we show the malignancy score of each frame in a BUS sequence of a benign case. Ideally, the malignancy score of each frame should be lower than 0.5 as we have a benign tumor. However, as shown, some BUS frames such as frame 2 and frame 13 obtain a malignancy score higher than 0.5. Hence, for such two frames, if fed into a CAD system that relies on a single image, a wrong classification will be obtained.

**Figure 6:**
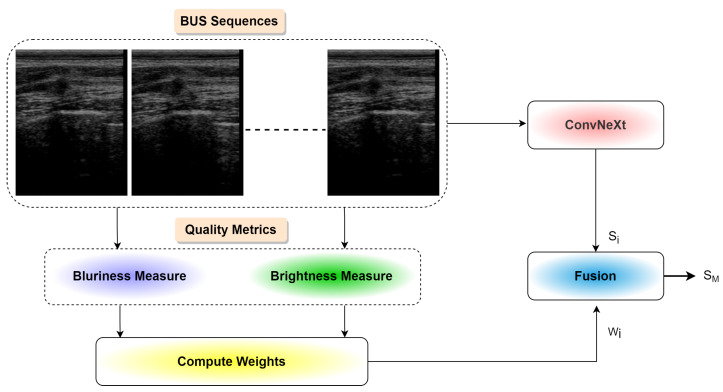
Schematic diagram of malignancy score pooling mechanism.

In this paper, we propose to classify benign and malignant breast tumors based on BUS sequences instead of single BUS images. In particular, we extracted radiomic features based on ConvNeXt from each frame in the BUS sequence and estimated the malignancy score of each frame. Figure 6 presents the step of the proposed malignancy score pooling mechanism. We calculated the malignancy score of the whole input BUS sequence as follows:

(5) $$S_{M}=\frac{1}{N_{q}}\sum w_{i}s_{i}$$

where $s_{i}$ is the malignancy score of frame *i*, $W=[w_{1},w_{2},\cdots ,w_{N}]$ is a weighting vector with a length *N*, where any element $w_{i}$ may hold 0 or 1. An element in *W* has a value of 1 if the quality of the BUS frame exceeds the thresholds of the brightness and blurriness scores, and $N_{q}$ is the number of the frames in the BUS sequence exceeding the thresholds of the brightness and blurriness scores.

**Blurriness score**: To estimate the blurriness score, a variance of the BUS image $I_{BUS}\left(p,q\right)$ intensity smoothed by a Gaussian filter $G_{f}\left(p,q\right)$ [27,28] was employed. The Gaussian filter can be expressed as follows:

(6) $$G_{f}\left(p,q\right)=\frac{1}{\left(2\pi \sigma^{2}\right)}e^{-\frac{\left(p^{2}+q^{2}\right)}{2\sigma^{2}}},$$

where *p* and *q* stands for the coordinates of an image $I_{BUS}\left(p,q\right)$, and σ stands for the standard deviation of the Gaussian distribution. A Laplacian operator showing the variation of the gradient ($\nabla I_{BUS}$) was estimated for two dimensions as a sum of the second partial derivatives in the Cartesian coordinates as follows:

(7) $$\nabla {}^{2}I_{BUS}\left(p,q\right)=\frac{\partial^{2}I_{BUS}}{\partial p^{2}}+\frac{\partial^{2}I_{BUS}}{\partial q^{2}},$$

A low score referred to a blurry image, and a high value confirmed the BUS was sharp based on the measured variation.

**Brightness–Darkness score**: Estimating brightness or darkness (due to the presence of shadows) can help identify distinct image properties. Here, we used the brightness estimation algorithm proposed in [29].

**Figure 7:**
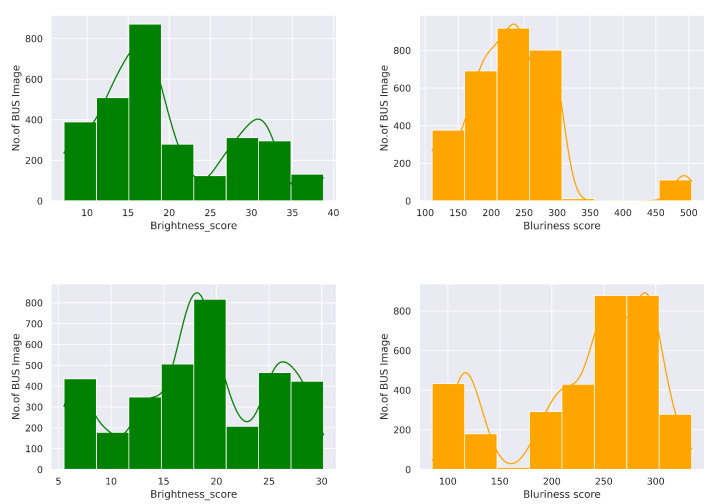
Brightness and blurriness scores analysis on the BUS sequence dataset. Top and below rows represent the benign and malignant classes, respectively. These plots were generated using the Python seaborn https://seaborn.pydata.org/ (accessed on 1 April 2022) and matplotlib https://matplotlib.org/ (accessed on 1 April 2022) libraries.

Figure 7 shows the brightness and blurriness scores analysis on the BUS sequence dataset. Specifically, we removed the BUS frames from two tails that obtained lower quality metrics scores. We selected the range from 10 to 30 for both benign and malignant classes in terms of brightness score. However, we ignored samples with blurriness scores less than 200 and greater or equal to 300. The main reason for selecting a brightness score in the range of 10 to 30 was to avoid the artifacts in BUS images. Shadows come to a darker contrast that may confuse the deep learning model for predicting benign and malignant tumors. To handle this issue, we set the lower brightness limit to 10. Moreover, the higher gain or amplification can degrade the BUS image details and could be the cause of imaging artifacts. To overcome this challenge, we chose to exclude the BUS image with a brightness score of more than 30. In turn, blurriness is generally caused by the motion during image acquisition by the sonographer, or other factors may be involved in it. However, blurriness could hinder the image properties in BUS imaging. The selected range was taken by computing the minimum, maximum, and average values across all the samples. Based on the curve presented in Figure 7, the highlighted peak where the majority of the BUS images fitted in the range of 200 to 300 provides evidence for determining the optimum value. It shows that images in this range are sharp.

### 3.3. Visual Explanation and Interpretation

This paper employed different techniques to produce visual explanations for the proposed ConvNeXt-based radiomics system’s decisions to make them explainable. Specifically, we utilized the Grad-CAM method (i.e., gradient-weighted class activation mapping) and presented the overall malignancy score overlaid on the BUS images. The Grad-CAM technique employs the gradients of any target class, e.g., malignant tumor in our ConvNeXt-based radiomics network, streaming into the last Conv layer to create a localization map emphasizing the vital regions in the input image that participate in the prediction of the class [30].

Assuming that we have a class $c,y^{c}$, the score for this class before the softmax with regard to the feature map activations $A^{k}$ of a Conv layer, i.e., $\frac{\partial y^{c}}{\partial A^{k}}$, was calculated to produce the Grad-CAM localization map $M_{\mathrm{Grad}-\mathrm{CAM}\;}^{c}\in R^{u\times v}$, where *u* and *v* are the width and height of the Grad-CAM localization map. The neuron importance weights $\beta_{k}^{c}$ were calculated by applying a global-average-pooling on the gradients flowing back over the width *i* and height *j* as follows [30]:

(8) $$\beta_{k}^{c}=\frac{1}{Z}\sum_{i}\sum_{j}\;\frac{\partial y^{c}}{\partial A_{ij}^{k}}$$

Then, the neuron importance weights $\beta_{k}^{c}$ were used to produce a weighted combination of forward activation maps as follows [30]:

(9) $$M_{\mathrm{Grad}-\mathrm{CAM}}^{c}=\mathrm{ReLU}\overset{\mathrm{linear}\;\mathrm{combination}\;}{\overset{︷}{\left(\sum_{k}\beta_{k}^{c}A^{k}\right)}}$$

ReLU was used to highlight the features that positively impacted the target class *c*. In other words, ReLU was employed here to highlight the pixels whose intensity must be raised to boost a differentiable activation $y^{c}$.

**Figure 8:**
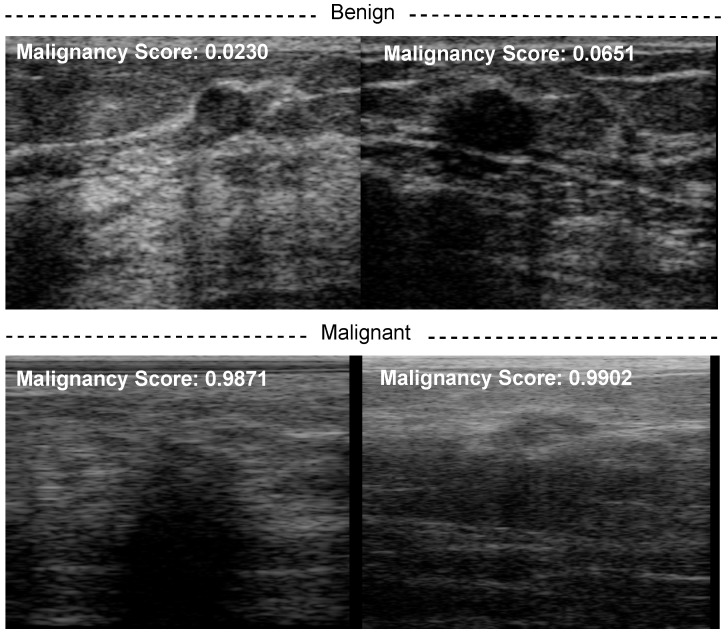
Examples of classification results using our method: (**top**) benign and (**bottom**) malignant breast masses.

Furthermore, we also computed the malignancy score of each BUS sequence and overlaid it on the BUS images as shown in Figure 8.

### 3.4. Evaluation Metrics

In this study, the performance of the proposed approach was assessed using different evaluation metrics, namely, accuracy, precision, recall, and F1-score. These metrics can be defined as follows [31]:

(10) $$\mathrm{Accuracy}=\frac{TP+TN}{P+N}$$

(11) $$\mathrm{Precision}=\frac{TP}{TP+FP}$$

(12) $$\mathrm{Recall}=\frac{TP}{TP+FN}$$

(13) $$F1-\mathrm{score}=\frac{TP}{TP+\frac{1}{2}\left(FP+FN\right)}$$

In these expressions, *TP* stands for the number of malignant BUS sequences correctly classified as malignant; *TN* stands for the number of benign BUS sequences correctly classified as benign; *FP* stands for the number of benign BUS sequences wrongly classified as malignant; *FN* stands for the number of malignant BUS sequences wrongly classified as benign.

### 3.5. Dataset

A database of 31 malignant and 28 benign BUS sequences was used to build and evaluate the proposed CAD system, where each BUS sequence corresponded to a patient. This dataset is part of a clinical database of ultrasonic radiofrequency strain imaging data created by the Engineering Department of Cambridge University. The number of ultrasound images in the benign and malignant BUS sequences was 3911 and 5245, respectively. It should be noted that we employed data augmentation techniques including horizontal flipping with probability 0.5, rotating of 90 degrees, scaling with 0.2, median blurring, and contrast-limited adaptive histogram equalization (including a clip limit equal to 4.0, and a tile grid of size 8×8) to increase the number of training samples. After the data augmentation step, we generated more than thirty thousand BUS images consisting of benign and malignant tumors.

## 4. Results and Discussion

In this subsection, we present and discuss the results of the experiments listed below:

- Performance analysis of the SUI CAD system based on different CNN networks and vision transformers.
- Performance analysis of the proposed method based on BUS sequences, ConvNeXt radiomics, and the malignancy score pooling mechanism.

The results of the proposed radiomics approach, including alternative CNN-based and transformer-based radiomics approaches, are shown in this subsection. Table 2 presents the performance of different deep CNN-based radiomic features extracted from a a single BUS image to differentiate between benign and malignant tumors (i.e., SUI CAD systems). Specifically, we employed EfficientNetV2 [32], EfficientNet-B7 [33], MobileNetV3 [34], and ResNet-101 [12] to classify breast tumors as benign or malignant. We employed pretrained models and fine-tuned them with BUS data. As one can see in Table 2, MobileNetV3-based radiomics outperforms EfficientNetV2, EfficientNet-B7, and ResNet-101. It achieves an accuracy of 88.17%, precision of 88.60%, recall of 86.60%, and F1-score of 87.28%. EfficientNetV2- and EfficientNet-B7-based radiomics obtain similar results with an accuracy lower than 85%. ResNet-101 achieves the second-best classification results, where it obtains an accuracy rate 2–3% higher than EfficientNetV2- and EfficientNet-B7-based radiomics. The F1-score of MobileNetV3 is 3% higher than ResNet-101. As a result, MobileNetV3 CNN may be a proper model to predict breast tumor malignancy scores from a single BUS image.

Table 3 presents the breast tumor classification results of different vision-transformer-based radiomic features extracted from a single BUS image. Here, vision transformer (ViT) [35], ResMLP [36], Swin Transformer [37], and ConvNeXt [15] were employed. ConvNeXt outperforms all other transformers in classifying breast tumors with accuracy, precision, recall, and F1-score higher than 88%. ResMLP obtains an accuracy of 86.16%, which is 2% lower than ConvNeXt. ViT provides an accuracy much lower than other transformers. ConvNeXt also outperforms the results of all CNNs (EfficientNetV2, EfficientNet-B7, MobileNetV3, and ResNet-101) mentioned in Table 2. We selected ConvNeXt to extract radiomic features from BUS sequences to compute the malignancy score based on this analysis.

**Figure 9:**
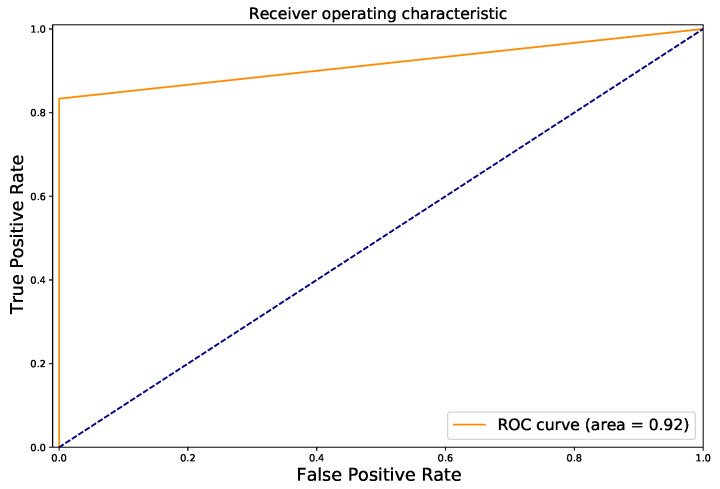
ROC curve of the proposed method.

Table 4 shows the results of the proposed approach, in which ConvNeXt was used to extract radiomic features from BUS frames and predict the malignancy score from each frame. The proposed method achieves an accuracy, precision, recall and F1-score higher than 91%. As one can see, the proposed method outperforms all SUI BUS CAD system discussed in Table 2 and Table 3. The F1-score of the proposed approach is 4% higher than the SUI CAD system based on ConvNeXt. We also replaced the ConvNeXt network with MobileNetV3 (best CNN in Table 2) in the proposed method, finding that it obtains an accuracy of 87.42%, which is much lower than that of ConvNeXt. Figure 9 shows the ROC curve of the proposed method. As one can see, we achieve an AUC value of 0.92, which is much higher than the MobileNetV3 (0.88).

**Figure 10:**
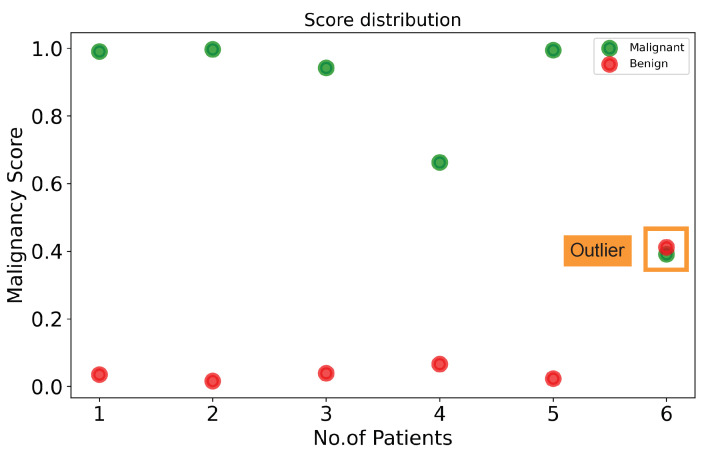
Illustration of patient-wise malignancy analysis.

Figure 10 visualizes the malignancy scores of the proposed method with BUS sequences of some patients. Given a BUS sequence fed to our method, if the malignancy score is higher than or equal to a threshold, the tumor is classified as malignant. If the malignancy score is lower than a threshold, the cancer is classified as benign. In our study, a threshold of 0.5 was used. As shown in Figure 10, five benign cases (marked by red dots) have low malignancy scores (<0.1), while four malignant cases (marked by green dots) have malignancy scores higher than 0.85. There are some outliers: one misclassified BUS sequence (marked by a square). An interesting observation is the malignancy scores of the misclassified BUS sequence are close to the threshold of 0.5. It should be noted that the threshold used to map probabilities to class labels can also be tuned to find the optimal value using a grid search algorithm, thus reducing the number of misclassified BUS sequences.

Figure 8 shows the malignancy scores of two BUS sequences having benign tumors (first row) and two BUS sequences having malignant tumors (second row). It should be noted that the malignancy score ranges from 0 to 1, where a malignancy score of 0 stands for no malignancy (i.e., benign tumors). As the malignancy score value approaches 1, the malignancy of the tumor increases. The proposed method obtains very low malignancy scores of 0.023 and 0.0651 for the two benign tumors. In turn, the proposed method produces high malignancy scores for the two malignant tumors.

**Figure 11:**
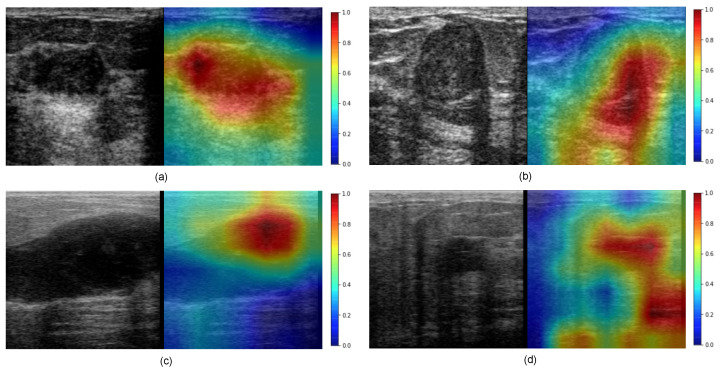
Visualization of activation maps using Grad-CAM for (**a**,**b**) benign, and (**c**,**d**) malignant cases.

Figure 11 presents a visual interpretation of the proposed method using the Grad-CAM technique as explained in Section 3.3. As one can see, the pixels that highly contribute to the decisions of ConvNeXt (i.e., classifying tumors as benign or malignant) are highlighted in red, while the pixels that have a very low contribution to the decisions of ConvNeXt are highlighted in blue color. In the case of BUS images shown in Figure 11a–c, the red color in the two heatmaps is concentrated around the region of the tumors. In turn, in Figure 11d the red color appears in a shadow region as well as in the tumor region.

**Figure 12:**
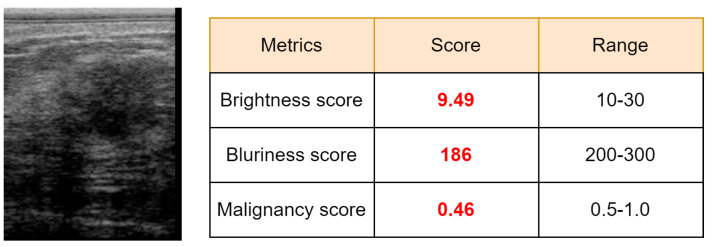
Malignant sample misclassified by our method.

Indeed, the existence of dense breast fat and glandular tissue induces extended attenuation of the sent ultrasound energy; this attenuation is additionally combined through the inherent depth and frequency-dependent attenuation that affect waves in the ultrasonic settings. Because of this, the ultrasound image quality is reduced by high attenuation. This process results in a poor contrast-to-noise ratio (CNR) and SNR. This poor image quality creates issues for a clinician to precisely diagnose and curate them. Figure 10 shows the malignancy score analysis at the patient level. Each class includes six patients. A higher malignancy score confirms the patient’s tumor is malignant, otherwise it is benign. Figure 12 presents a malignant sample wrongly misclassified as benign, which produced a low malignancy score (0.46). As we can see, this BUS image has a limited quality as the brightness score (9.49) and the blurriness score (186) are lower than the predefined minimum thresholds, 10 for brightness and 200 for blurriness.

Based on a visual inspection of BUS sequences, a few intermediate frames attained higher scores due to better image quality. However, lower score frames have shadows, speckle noise, brightness, darkness, and blurriness caused by the motion of the sonographer or patient during image acquisition. Hence, the BUS image quality was considered when calculating the overall malignancy score of the input BUS sequence as explained in Section 3.2. As a result, the proposed method achieved an accuracy and F1-score higher than 91%.

In turn, breast tumor classification results could be improved if an efficient ultrasound image enhancement mechanism as in [38,39] was integrated with the proposed CAD system. Image enhancement can handle the problem mentioned above, improve the quality of BUS images, and improve the classification rate. This point will be considered in our future work. It should be noted that the proposed method completely works in an end-to-end manner as it does not need any preprocessing (e.g., ROI selection). In future work, we will consider using ROIs instead of the entire BUS image to further enhance the classification results.

## 5. Conclusions

This paper proposed an efficient deep-learning-based radiomics method for predicting breast cancer malignancy from BUS sequences. The proposed method consisted of three main components: (1) a deep ConvNeXt network to extract robust radiomic features to predict the malignancy score of breast tumors, (2) a pooling mechanism to generate the malignancy score of each input BUS sequence, and (3) a visual explanation step to generate heat maps superimposed on ultrasound images that help interpret the deep learning model decisions. A BUS sequence dataset containing 31 malignant and 28 benign cases was used to assess the efficacy of the proposed method. Our experiments also compared single ultrasound image CAD systems with our method considering different CNN networks (EfficientNetV2, EfficientNet-B7, MobileNetV3 and ResNet-101) and vision transformers (ViT, ResMLP and Swin transformer). The proposed method outperformed all single ultrasound image CAD systems. The proposed method achieved an accuracy and F1-score higher than 91%. Moreover, the F1-score of the proposed approach was 4% higher than the single ultrasound image CAD system based on ConvNeXt. We also demonstrated that the quality of the BUS images can affect the accuracy of the malignancy prediction models. We revealed that the proposed malignancy score pooling mechanism improved the classification accuracy as it ignored the low quality BUS images when calculating the final malignancy score.

Future work will be focused on validating the proposed method in another breast cancer dataset and with ultrasound sequences of other diseases such as thyroid cancer.

## Figures and Tables

**Table 1:** Summary of research for the diagnosis of breast cancer based on ultrasound images.

Study	Methods	Results	Dataset	Year
[16]	generic deep CNN and transfer learning	accuracy <90%	private SI BUS and thyroid dataset	2021
[17]	six traditional and CNNs SVM, KNN, DT, NB	76.10% accuracy	public SI breast dataset	2021
[18]	DarkNet53 model and fusion technique	99.1% accuracy	public SI BUS dataset	2022
[19]	R-CNN, faster R-CNN, YOLO SSD, AlexNet, ZFNet, VGG ResNet, GoogleNet, and DenseNet	87.5% accuracy	private SI BUS dataset	2019
[20]	segmentation-based attention and feature fusion	95.0% AUC	private SI BUS dataset	2022
[21]	multitask deep method	74.1% accuracy	private dataset of 3D ABUS	2021
[22]	handcrafted features, GBC RF, SVM, AdaBoost, and LR	97% accuracy	public SI BUS dataset	2021
[23]	semisupervised GAN, Inception-V3	90.4% accuracy	private SI BUS dataset	2021

**Table 2:** Results of SUI BUS CAD systems based on different CNN networks. The best results are highlighted in bold.

Model	Metrics			
	Accuracy (%)	Precision (%)	Recall (%)	F1-Score (%)
EfficientNetV2 [32]	83.09	85.31	79.70	81.04
EfficientNet-B7 [33]	82.30	85.94	78.24	79.71
MobileNetV3 [34]	**88.17**	**88.60**	**86.48**	**87.28**
ResNet-101 [32]	85.20	84.81	83.98	84.31

**Table 3:** Results of SUI BUS CAD systems based on different transformers and ConvNeXt. The best results are highlighted in bold.

Model	Metrics			
	Accuracy (%)	Precision (%)	Recall (%)	F1-Score (%)
ViT [35]	72.95	71.76	70.44	70.86
ResMLP [36]	86.16	85.82	84.81	85.24
Swin transformer [37]	80.24	81.62	76.68	77.89
ConvNeXt [15]	**88.90**	**88.21**	**88.77**	**88.46**

**Table 4:** Results of the proposed approach based on BUS sequences. The best results are highlighted in bold.

Model	Metrics			
	Accuracy (%)	Precision (%)	Recall (%)	F1-Score (%)
Proposed method	**91.66**	**93.05**	**92.69**	**92.33**
MobileNetV3	87.42	88.54	88.80	88.67

## Data Availability

The BUS dataset used in this study is part of a clinical database of ultrasonic radiofrequency strain imaging data created by the Engineering Department of Cambridge University http://mi.eng.cam.ac.uk/research/projects/elasprj/ (accessed on 1 April 2022).
